# FLIM as a Promising Tool for Cancer Diagnosis and Treatment Monitoring

**DOI:** 10.1007/s40820-021-00653-z

**Published:** 2021-06-03

**Authors:** Yuzhen Ouyang, Yanping Liu, Zhiming M. Wang, Zongwen Liu, Minghua Wu

**Affiliations:** 1 Hunan Provincial Tumor Hospital and the Affiliated Tumor Hospital of Xiangya Medical School, Central South University, Changsha, 410013 Hunan People’s Republic of China; 2 School of Physics and Electronics, Hunan Key Laboratory for Super-Microstructure and Ultrafast Process, Central South University, 932 South Lushan Road, Changsha, 410083 Hunan People’s Republic of China; 3 The Key Laboratory of Carcinogenesis of the Chinese Ministry of Health, The Key Laboratory of Carcinogenesis and Cancer Invasion of the Chinese Ministry of Education, Cancer Research Institute, Central South University, Changsha, 410008 Hunan People’s Republic of China; 4 Shenzhen Research Institute of Central South University, A510a, Virtual University Building, Nanshan District, Southern District, High-tech Industrial Park, Yuehai Street, Shenzhen, People’s Republic of China; 5 State Key Laboratory of High-Performance Complex Manufacturing, Central South University, 932 South Lushan Road, Changsha, 410083 Hunan People’s Republic of China; 6 Institute of Fundamental and Frontier Sciences, University of Electronic Science and Technology of China, Chengdu, 610054 Sichuan People’s Republic of China; 7 School of Chemical and Biomolecular Engineering, The University of Sydney, Sydney, NSW 2006 Australia

**Keywords:** Fluorescence lifetime imaging microscopy, Förster resonance energy transfer, Reduced form of nicotinamide adenine dinucleotide, Biosensors, Cancer

## Abstract

Fluorescence lifetime imaging microscopy (FLIM) has been rapidly developed over the past 30 years and widely applied in biomedical engineering. Recent progress in fluorophore-dyed probe design has widened the application prospects of fluorescence. Because fluorescence lifetime is sensitive to microenvironments and molecule alterations, FLIM is promising for the detection of pathological conditions. Current cancer-related FLIM applications can be divided into three main categories: (i) FLIM with autofluorescence molecules in or out of a cell, especially with reduced form of nicotinamide adenine dinucleotide, and flavin adenine dinucleotide for cellular metabolism research; (ii) FLIM with Förster resonance energy transfer for monitoring protein interactions; and (iii) FLIM with fluorophore-dyed probes for specific aberration detection. Advancements in nanomaterial production and efficient calculation systems, as well as novel cancer biomarker discoveries, have promoted FLIM optimization, offering more opportunities for medical research and applications to cancer diagnosis and treatment monitoring. This review summarizes cutting-edge researches from 2015 to 2020 on cancer-related FLIM applications and the potential of FLIM for future cancer diagnosis methods and anti-cancer therapy development. We also highlight current challenges and provide perspectives for further investigation.

## Highlights

- Fluorescence lifetime imaging microscopy (FLIM) applications for cancer diagnosis and treatment monitoring combined with reduced form of nicotinamide adenine dinucleotide, Förster resonance energy transfer (FRET), and biosensors are reviewed.
- Principles of FLIM, previous clinical applications, and development history are introduced.
- The current challenges and prospects for the potential of FLIM for cancer diagnosis and promotion of the effect of anti-cancer treatment are discussed.

## Graphical abstract

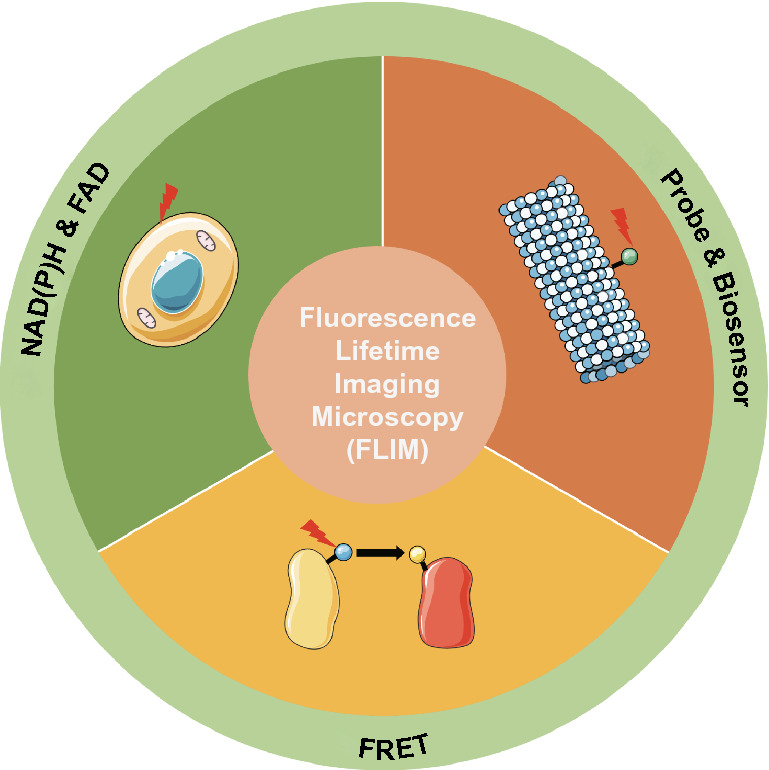

## Introduction

Fluorescence has become an essential tool for scientific research especially following the discovery of fluorescent proteins in the last century because of its biocompatibility [1]. Multiple characteristic parameters of fluorescence can be applied for constructing images, including intensity, spectrum, and lifetime. Considering the advantages of fluorescence lifetime, such as high sensitivity to molecular conformation and environment changes, as well as the ability distinguishing molecules with overlapping spectra, fluorescence lifetime, an intrinsic property of fluorescent molecule, is promising for biomedical applications [2, 3].

To accurately detect fluorescence lifetime, fluorescence lifetime imaging microscopy (FLIM) has been continuously improved over the decades, which can be summarized as the imaging speed, the accuracy of fluorescence lifetime measurements, and the imaging resolution improvement [3]. Through the innovative union of the ultrashort laser with laser scanning, the first time-correlated single photon counting (TCSPC)-FLIM was born in the 1980s. However, because of the large photon requirement of TCSPC-FLIM for accurate lifetime measurements, imaging speed is one of its shortcomings. The wide-field imaging, single-photon avalanche diode (SPAD) arrays, improved scanning methods, and better fitting algorithms all can accelerate imaging and numerous fast FLIM techniques have been reviewed in detail [4, 5]. Besides, after the TCSPC-FLIM invention, some other problems, including depth penetration restriction, background fluorescence interference, photo-bleaching, also impeded wider clinical applications of FLIM such as fluorescence lifetime detection in thick samples. Therefore, FLIM resolution and lifetime measurement accuracy improvement are the issues attracting a great number of researchers’ attention. For this purpose, the two-photon FLIM (TP-FLIM) technique is a promising method. It is implemented by using two longwave photons to excite a fluorescence molecule, leading to the emission of shortwave photons at high intensity. From 1990 to 2010, the first two-photon microscope, the first two-photon application on human skin, and the first clinical two-photon time-resolved imaging were reported in succession. Moreover, based on material innovations, the applications of TP-FLIM imaging can be further extended by thermally activated delayed-fluorescence fluorophores with prolonged fluorescence lifetimes and high two-photon absorption efficiencies [6, 7]. Recently, various applications of TP-FLIM, including digestive tract tumor diagnosis, have also been reviewed, proving the clinical application potential of TP-FLIM [8, 9]. Furthermore, the multiphoton FLIM method has also been developed since the first successful application of clinical multiphoton tomography (MPT) in 2002. Later, clinical multiphoton fluorescence endoscopy was also successfully constructed [10]. Through its continuous development, nowadays, numerous multiphoton FLIM tomographs such as MPTcompact and DermaInspect are already in clinical use in many countries [11].

**Fig. 1:**
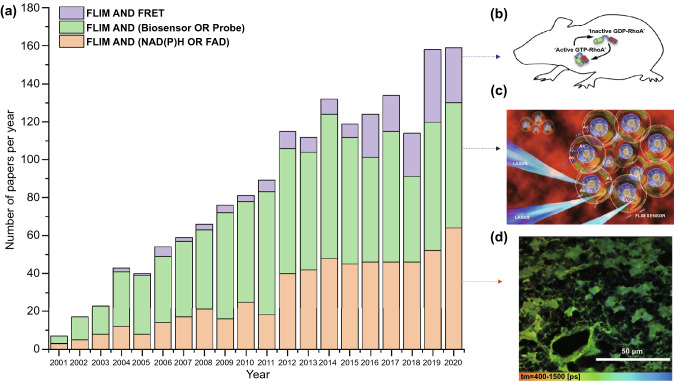
Numbers of publications per year in the field of FLIM with NAD(P)H or FAD (orange), probes or biosensors (green), and FRET (purple) from January of 2001 to December of 2020 (data from the web of science, January 2021). **a** Histogram of the number of papers in each field from 2001 to 2020; **b** A RhoA-FRET biosensor mouse was used to depict and quantify RhoA activity spatiotemporal distributions in native mammalian tissues in vivo during development and disease progression. Reproduced with permission from Ref. [80]. Copyright 2017, Elsevier; **c** Under laser excitation, a peptide biosensor with TCSPC-FLIM was utilized to monitor acetylation activity in real time. Reproduced with permission from Ref. [106]. Copyright 2019, American Chemical Society; **d** Representative color-coded image of NADH lifetimes in GSC tumor cells. Reproduced with permission from Ref. [36]. Copyright 2020, The International Society for Optical Engineering-SPIE. (Color figure online)

Based on the continuous development of these instruments and analysis methods, the range of FLIM applications has been broadened in biomedical research. Over the past few decades, this technique has been used for the detection of inter-protein interactions, calcium concentrations, and other applications [12, 13]. As for clinical medicine, FLIM has been applied to inflammation site detection, dermatosis differential diagnosis, tumor boundary delineation, etc. Notably, among these research fields, cancer diagnosis and treatment has attracted significant attention based on the high incidence and mortality of cancer, which necessitate early cancer diagnosis and anti-cancer drug efficiency enhancement. FLIM is a promising technique for such applications. Therefore, rather than to comprehensively summarize the FLIM techniques development or its extensive applications in numerous research fields, including cosmetic products and neuroscience which are already available elsewhere, this review mainly introduces the FLIM applications related to cancer diagnosis and anti-cancer treatment improvement in three main research areas: (i) FLIM with autofluorescence coenzymes for monitoring cell metabolism, (ii) FLIM in combination with Förster resonance energy transfer (FRET) pairs for detecting molecule interactions, and (iii) FLIM with well-designed probes for detecting intracellular or extracellular environments. In Fig. 1, it is evident that the number of publications on FLIM is increasing annually, which highlights the enormous potential of FLIM in various fields.

## Principles of FLIM

**Fig. 2:**
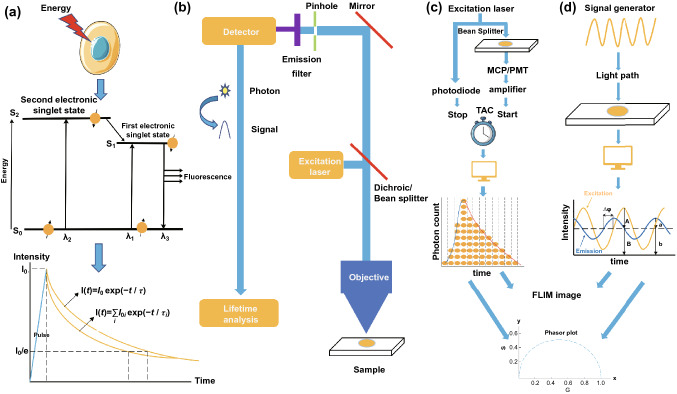
Basic principles of fluorescence lifetime, fluorescence imaging, TCSPC-FLIM, and FD-FLIM. **a** Principles of fluorescence lifetime. **b** Basic setup for fluorescence imaging with a lifetime analysis instrument. **c** TCSPC-FLIM system scheme. **d** FD-FLIM sketch map

As shown in Fig. 2a, the basic principle of fluorescence is that when a fluorophore is excited to a higher energy state by absorbing one or more photons, it returns to its ground state through a radiative or non-radiative process. Fluorescence emission is a radiative process, which can be recorded by an apparatus, and the fluorescence decay curve can be fitted by the algorithm [14, 15]. There are two rates determining the fluorescence lifetime (τ): The radiative decay rate *Γ* and the nonradiative decay rate *k. Γ* is based on the fluorophore electronic properties, whereas, *k* is relevant to the molecule interaction, FRET, etc. Under the simplest condition, fluorescence decay can be fitted as following exponential decay:

1 $$\frac{\mathrm{d}N(t)}{\mathrm{d}t}=-\left(\Gamma +k\right)N(t)$$

2 $$N\left(t\right)={N}_{0 }{e}^{-(\Gamma +k)t}={N}_{0}{e}^{-t/\uptau }$$

The *N*(0) and *N*(*t*) are the initial number of fluorescent molecules and the fluorophore population on the first singlet state, respectively. Therefore, the fluorescence lifetime is:

3 $$\tau =\frac{1}{\Gamma +k}$$

which indicates the average time fluorophores on the excited state before returning to the ground state. If a fluorescence decay curve is constructed as the chart in Fig. 2a, the fluorescence lifetime can be calculated as the time when the intensity decreases to 1/e of the initial intensity.

As for the FLIM apparatus, the basic setup for fluorescence imaging is presented in Fig. 2b. In FLIM, single-photon detectors and photon timing techniques are incorporated into this setup for lifetime measurement [16]. Single-photon detectors in FLIM are typically based on photon amplification through electrical current generation when photons impact a photovoltaic such as the photomultiplier tube (PMT) or avalanche photodiode (APD). The photon timing apparatus then transforms the photon signal into a digital signal for further lifetime analysis, such as leading-edge and constant fraction discriminator (CFD) analysis, time-to-amplitude conversion (TAC), analog-to-digital conversion (ADC), time-to-digital conversion (TDC), and streak camera analysis.

As shown in Table 1, FLIM setups are diverse because FLIM depicts the fluorescence lifetime τ in both the time domain (TD) and frequency domain (FD) [17]. TD-FLIM includes streak cameras, the time gate technique, and TCSPC, whose principles have been introduced previously [18]. Among these TD methods, the most prevalent technique is TCSPC [19]. The basic principles of TCSPC can be briefly illustrated as the scheme in Fig. 2c. The laser will firstly be separated into two beams, one of which enters into the imaging system to excite sample to produce a fluorescence signal. The fluorescence signal will be recorded by the micro-channel-plate/photomultiplier (MCP/PMT) and be amplified by the amplifier to generate a start signal for TAC. While the other laser passes a fast photodiode to produce a stop signal for TAC system. After the TAC and ADC signal conversions, photons are classified into different time channels based on the arrival time. Through computer processing, a fluorescence decay curve is then generated, and the fluorescence lifetime of each pixel is used to perform FLIM imaging. Besides, the time-gated method is based on the detector, such as the charge-coupled device camera (CCD), to record fluorescence decay at different time gates for reconstructing the exponential fluorescence decay curve [20]. The streak camera method mainly consists of a streak camera, a pulse laser, and a picker. Through scanning electric field accelerations, photoelectrons arriving at different time will be separated in space. The fluorescence lifetime can be obtained by analyzing the resulting spatial distribution [21].

**Table 1:** Time domain & Frequency domain FLIM

Method		Excitation laser	Detector	Imaging method	Advantages	Shortcomings	Refs.
Time domain	TCSPC	Pulsed laser	PMT APD/SPAD Hybrid detector	Scanning imaging	High- resolution Reduced background blur Most widely used Accurate measurement Low photodamage and photobleaching	Slow imaging speed High requirement of hardware and software	[3, 16–19]
	Time gate	Pulsed laser	CCD/ICCD PMT	Wide-field imaging	Fast imaging speed Low photodamage Convenient spatial information acquisition	High-resolution demand for instruments Poor accuracy of lifetime measurement Unsuitable for multicomponent measurement Scarce optical sectioning capability	[3, 17, 18, 20]
	Streak camera	Pulsed laser	Streak camera	Multidimensional Scanning imaging	High-resolution Low photodamage Fast imaging speed	High cost High technical demanding	[16, 17, 21]
Frequency domain		Sinusoidally modulated light/Pulsed laser (High repetition rate)/Continous wave (CW)	CCD	Scanning/Wide-field imaging	Low requirement of hardware and software Fast imaging speed (wide imaging) Low photodamage and photobleaching	High photodamage Background interference (Wide-field imaging) Limited photon timing resolution (Wide-field) Restricted imaging speed (scanning imaging) and imaging depth (wide-field imaging)	[3, 16–18]

*PMT* photomultiplier tube, *APD* avalanche photodiode, *CCD* charge-coupled device, *ICCD*: intensified charge-coupled device

FD-FLIM is based on modulation technology for generating a sinusoid. As shown in Fig. 2d, the signal generator first generates a sine-wave pulse that will travel through a predefined light pathway to excite the sample. The emission pulse wave is recorded by instruments, and based on computer processing, an emission wave is generated, as shown in Fig. 2d. Analyzing the change in demodulation represented by the *M* (counted by Eq. (4)) and the phase delay △*φ*, the fluorescence lifetime (fluorescence module lifetime (*τ*_*M*_) and phase shift lifetime (*τ*_△φ_)) can be calculated directly using Eqs. (5) and (6).

4 $$\mathrm{M}= \frac{a/b}{A/B}$$

5 $$\tau_{\rm M}=\frac{1}{\omega }\sqrt{\frac{1}{{M}^{2}}-1}$$

6 $$ \tau_{\Delta \varphi} = \frac{{\tan \left( {\Delta \varphi } \right)}}{\omega } $$

To fit the fluorescence decay curves, in the fitting analysis method, two types of exponential decays are commonly used. Equation (7) is used for single-exponential analysis, whereas Eq. (8) is used for multi-exponential decay. Multi-exponential decay is suitable for complex fluorescence lifetime decay, particularly for the coexistence of different fluorophores.

7 $$\mathrm{I}\left(\mathrm{t}\right)={\mathrm{I}}_{0}\mathrm{exp}\left(-\frac{\mathrm{t}}{\uptau }\right)$$

8 $$I\left(t\right)=\sum_{i}{I}_{oi}\mathrm{exp}\left(-\frac{t}{{\tau }_{i}}\right)$$

(*I*_0_: intensity at time *t* = 0; *I*_0i_: intensity at *t* = 0 of each lifetime component; τ_i_: fluorescence lifetime of each lifetime component).

As these two methods require the estimation of molecular species numbers of components, the fluorescence lifetime analysis is sometimes not accurate and convenient. A promising non-fitting analysis method the phasor analysis, was put forward, which avoids assuming mono- or multi-exponential model to fit the data and is different in TD-FLIM and FD-FLIM [22]. In TD-FLIM, the fluorescence decays at each pixel *F*(t) can be transformed into cosine (g) and sine (s) coordinates in the phasor plot using Eqs. (9) and (10).

9 $$g = \frac{{\int }_{0}^{\infty }F(t)\mathrm{cos}(\omega t)\mathrm{d}t}{{\int }_{0}^{\infty }F(t)dt}$$

10 $$s = \frac{{\int }_{0}^{\infty }F(t)\mathrm{sin}(\omega t)\mathrm{d}t}{{\int }_{0}^{\infty }F(t)\mathrm{d}t}$$

where ω is the laser repetition angular frequency

If *F*(t) is a single exponential decay, g and s can be calculated directly using Eqs. (11) and (12).

11 $$g=\frac{1}{1+{(\omega \tau )}^{2}}$$

12 $$s=\frac{(\omega \tau )}{1+{(\omega \tau )}^{2}}$$

As for the phasor analysis in FD-FLIM, the demodulation M and phase delay △φ are transformed into g and s coordinates using Eqs. (13) and (14) to represent a phasor vector on the plot.

13 $$g=M* \cos \left(\Delta {\varphi }\right)$$

14 $$s=M*\sin\left(\Delta {\varphi }\right)$$

Based on the phasor vector locations or clustering on the phasor plot, FLIM data can be analyzed according to several different point distribution types in the phasor plot. [23, 24]. For instance, a universal circle will be firstly constructed by *s* = (*g*(1−*g*))^1/2^. The phasors of pixels with single-exponential or multi-exponential decay profiles will locate on or in the semi-circle, respectively. If there are two lifetime components, a “tie line” will connect the phasor locations of the two molecular species, where the relative positions of points on this line represent the relative contribution of these two species. In this manner, the molecular species number is easily ascertained, and color-coded FLIM can be performed.

## FLIM Combined with NAD(P)H for Cancer Metabolism Monitoring

The reduced form of nicotinamide-adenine dinucleotide (i.e., NADH), nicotinamide-adenine dinucleotide phosphate (i.e., NADPH), and flavin adenine dinucleotide (i.e., FAD) are potent for metabolism monitoring based on their intrinsic fluorescence and the nonfluorescent characteristic of the reduced form of FAD (i.e., FADH_2_) and NAD. Besides, because of the identical spectral characteristics of NADH and NADPH, the acronym "NAD(P)H" is frequently used [25].

The clinical applications of optical redox ratios, which can be calculated as FAD/[NAD(P)H + FAD], for oral and cervical cancer diagnosis have been discussed [26, 27]. However, due to the low quantum yield of NAD(P)H and FAD, the intensity-based method relying on optical redox ratios, is not as sensitive as we expect because of the background fluorescence interference. The fluorescence lifetime τ may hold promise for solving this deficiency. For lifetime measurement in NAD(P)H-FLIM research, bi-exponential decay is frequently applied to distinguish free and protein-bound cofactors [28]. The mean fluorescence lifetime τ_m_ can be calculated using Eq. (12), and the fluorescence lifetime of free NAD(P)H is shorter than that of protein-bound NAD(P)H, whereas free FAD possesses a longer fluorescence lifetime than protein-bound FAD [29].

12 $$ \tau_{m} = a_{1} \tau_{1} + a_{2} \tau_{2} , $$

where *τ*_1_ and *τ*_2_ are the lifetime of short- and long-lifetime components with *a*_1_ and *a*_2_ as their relative contributions to the whole fluorescence.

**Fig. 3:**
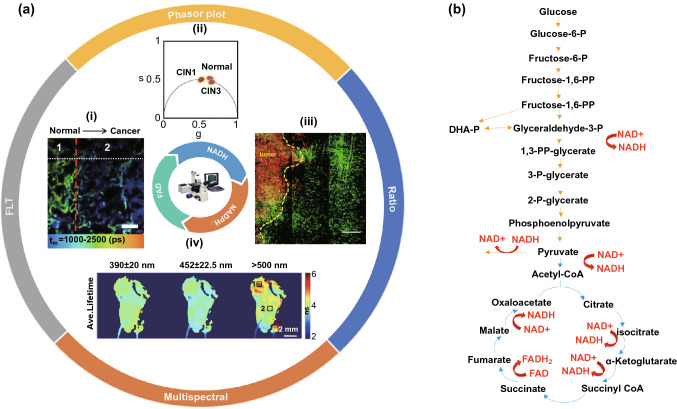
An overview of the autofluorescence of NAD(P)H and FAD in FLIM applications to cancer research and NADH, FAD in core glucose metabolim (Glycolysis, TCA). **a-i** Fluorescence lifetime assists lung tumor resection. A clear fluorescence lifetime decrease is exhibited from the normal tissue to the cancer region. Reproduced with permission from Ref. [31]. Copyright 2017, Elsevier; **a-ii** Phasor FLIM plot is utilized for distinguishing the normal cervical tissue sample, CIN I sample, and CIN II sample. Reproduced with permission from Ref. [56]. Copyright 2020, Optical Society of America; **a-iii** NADH/NAD + ratio is applied for ascertaining tumor fronts. A clear higher NADH/NAD + ratio is exhibited in tumor cells (red) compared with CX3CR1^+/gfp^ monocyte cells (green). Reproduced with permission from Ref. [53]. Copyright 2020, Springer Nature; **a-iv** Fluorescence lifetime maps of ex vivo human oral biopsy by ms-FLIM. Region 1 and region 2 marked in the fluorescence lifetime map were diagnosed as superficial invasive squamous cell carcinoma and dysplasia, respectively. Reproduced with permission from Ref. [64]. Copyright 2014, Optical Society of America; **b** Scheme of NADH and FAD in TCA and glycolysis. Yellow arrows indicate glycolysis processes, and blue arrows represent the processes of TCA, whereas red arrows signify NADH or FAD participation. (DHA-P, dihydroxyacetone phosphate; CoA, coenzyme A). (Color figure online)

Intensity and lifetime parameters possess respective advantages for different types of studies. Cancerous metabolic laboratorial and clinical studies have increasingly adopted lifetime index under the assistance of FLIM for NAD(P)H and FAD metabolism alteration detection. Figure 3a presents a summary of this section.

### Fluorescence Lifetime

As shown in Fig. 3b, glycolysis and tricarboxylic acid cycle (TCA) with oxidative phosphorylation (OXPHOS) are basic metabolic processes in which NADH and the reduced form of flavin adenine dinucleotide (FADH_2_) are produced or consumed. Unlike normal cells, most cancer cells prefer glycolysis over OXPHOS as their glucose metabolism tends to produce less NADH and FADH_2_ as observed by Warburg in the last century [30]. Moreover, during OXPHOS, the produced NADH will bind to its coenzyme leading to its fluorescence lifetime increase from less than 0.4ns in free-NADH conformation to ~ 2 ns of its coenzyme-bound form. Therefore, the Warburg effect results in a significant decrease of fluorescence lifetime. For example, the average fluorescence lifetime in human lung cancer tissue is reduced, as indicated by the data in Fig. 3a-i [31]. The fluorescence lifetime in cancerous lung tissue is shorter than that in normal and tumor margin tissues, which can be applied to tumor resection. This reduction has also been verified in H-ras oncogene-transfected cancer cells (W31) and malignant thyroid tissue [32, 33]. Various explanations for this effect have been proposed, including the proliferating cells’ large requirements for nucleotides, amino acids, and lipids of cancer cells, as well as gene mutations [34]. Whereas, it should also be emphasized that the metabolism of some cancer cells is inconsistent with the Warburg effect, indicating that cancer cells may possess higher OXPHOS levels (contrary to the Warburg effect), including leukemia cells, glioblastoma stem cells (GSCs), and cerebrospinal fluid metastatic cells [35–37]. This abnormal phenomenon is common in cancer stem cells and metastatic cancer cells, and the deviation is relevant to different gene expressions and tumor microenvironments, such as hypoxia and lactic acidosis [38–41]. Additionally, the urgent energy needs, as opposed to huge substance requirements, for progression and metastasis in metastatic cancer cells may also lead to this abnormal effect. Overall, different cancer cells possess diverse metabolic profiles, indicating that simple therapies aimed at inhibiting tumor cell glycolysis or OXPHOS may not succeed and could even lead to easier occurrence. Fortunately, to evaluate cell apoptosis under specific treatments, FLIM has also been successfully applied to detect the metabolism change in non-tumorigenic cells (MCF10-A), non-invasive cancer cells (MCF-7), and invasive cancer cells (MDA-MB-231) based on the fluorescence lifetime change [42, 43]. Besides, under clinical conditions, the biopsy sample preservation conditions can be optimized using fluorescence lifetime monitoring [44]. For identifying gene mutation germlines, such as the BRCA mutation with the increased risk of developing breast cancer, fluorescence lifetime analysis is also helpful [45].

The fluorescence lifetimes of cancer-adjacent healthy tissues can also be applied to ascertain tumor boundaries, such as those of leiomyomas [46]. Additionally, a vital process called the reverse Warburg effect has been proposed. In this process, stromal cells produce energy-rich metabolites such as lactate through enhanced aerobic glycolysis, and cancer cells can convert these metabolites into pyruvate, providing fuel for the TCA cycle [47]. For example, disseminated breast tumor cells exhibit increased OXPHOS and increased fluorescence lifetimes compared to co-culture bone marrow stromal cells in the bone marrow niche [48]. However, not all tumor-stroma cell interactions conform to the reverse Warburg effect. One such exception is the metabolism in a HeLa-fibroblast co-cultivation system [49]. In addition to diagnostic applications, these observations have opened novel avenues for treatment.

Under some conditions, the long lifetime τ_2_ (bound-NADH lifetime) is more sensitive, as indicated by the metabolic changes in breast cancer cells under glucose deprivation conditions [50]. Additionally, under enzymatic activity changes in glycometabolism, when using 10 μM FX11-inhibiting lactate dehydrogenase (LDH) and 50 mM dichloroacetate-relieving pyruvate dehydrogenase kinases (PDK) inhibition on the PDH of the cancer cells, the lifetime change is more obvious in *τ*_2_ group. Therefore, *τ*_2_ is more sensitive for monitoring carbon diversions compared to other parameters in this experiment [51].

### Ratios and Phasor Plots

**Fig. 4:**
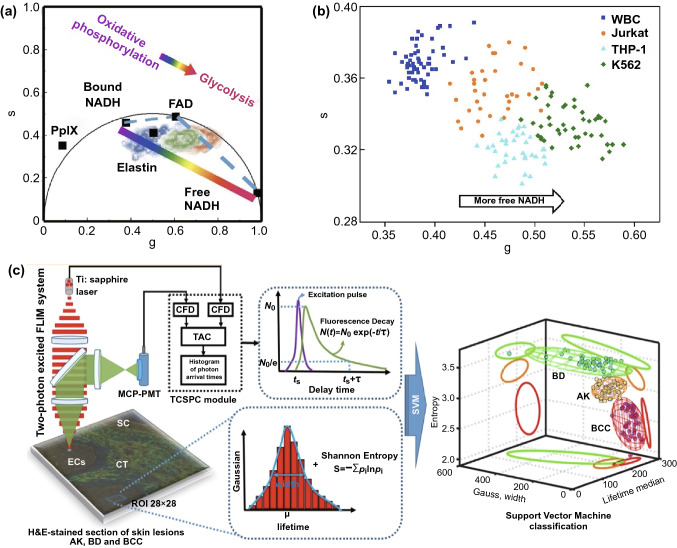
Phasor plot and multi-parameter classification system based on autofluorescence molecules. **a** Representative phasor plot based on autofluorescence molecules in the cell. Reproduced with permission from Ref. [56]. Copyright 2020, Optical Society of American; **b** Scatter plot of 65 WBCs (blue), 35 Jurkat cells (orange), 46 K562 cells (green), and 35 THP-1 cells (cyan) based on NADH fluorescence signals. Lower *s* values and higher *g* values represent additional free NADH, indicating glycolysis metabolism trend, similar to that in **a**. Reproduced with permission from Ref. [58]. Copyright 2020, Frontiers Media S.A.; **c** Schematic map of an SVM classification system for evaluating the stages of non-melanoma skin cancers. Reproduced with permission from Ref. [71]. Copyright 2019, American Chemical Society. (Color figure online)

As NADH and FAD change their existence forms during glycolysis or OXPHOS, the ratios such as NADH/NAD + and free/bound NADH, are also promising for cancer metabolism monitoring. NADH/NAD + oxidation–reduction pairs are crucial for electron transfer in mitochondria, and NADH/NAD + ratio is higher accompanied by enhanced anaerobic glycolysis [52]. As shown in Fig. 3a-iii, a higher NADH/NAD + ratio in tumor cells with a lower ratio in monocyte/macrophage cells can be applied for ascertaining tumor fronts, revealing that the fraction of anaerobic glycolysis in the tumor is larger than that in the surrounding monocytes or macrophages [53]. Besides, this NADH/NAD + ratio is relevant to free/bound NAD(P)H ratio based on previous researches, and the free/bound NAD(P)H ratio has been applied to melanoma progression detection [54]. Phasor plot is frequently applied for representing different free/bound-protein NAD(P)H ratios indicating alterations in cell metabolism under specific conditions, such as methionine stress or Wnt/β signaling inhibition [55]. As shown in Fig. 4a, FLIM phasor plot was constructed based on autofluorescence molecules such as free or bound NADH, elastin, and FAD. The colored lines between protein-bound and free NADH represent OXPHOS-to-glycolysis metabolism alteration. Furthermore, for clinical diagnosis, Phasor-FLIM has been successfully applied to cervical intraepithelial neoplasia (CIN) and neoplastic skin lesions, as shown in the phasor plot in Fig. 3a-ii [56]. This method is also adopted to glioblastoma multiforms (GBMs), leukemia subpopulation classification (Fig. 4b), as well as the identification of non-melanoma skin cancer (NMSC) [57–59]. Cancer metastasis is related to poor prognosis, and the original, first, and second metastatic cell lines (ZsG, LN1, LN2) can be distinguished by a free/bound NAD(P)H phasor plot [60]. Additionally, novel solutions for the chemo-resistance of cisplatin treatment have been proposed with the assistance from Phasor-FLIM [61]. Furthermore, a group of researchers introduced a novel redox ratio: Fluorescence Lifetime Redox Ratio (FLIRR), NAD(P)H-a_2_%(enzyme-bound)/FAD a_1_%(enzyme-bound). In the OXPHOS course, the NAD(P)H-a_2_%(enzyme-bound) fraction will increase and the FAD a_1_%(enzyme-bound) fraction will decrease, resulting in the increase of FLIRR, which is sensitive to the metabolism change in the cells. This ratio was successfully combined with NAD(P)H-Trp FRET efficiency to monitor tumor and stroma cell metabolism, and predict doxorubicin treatment response [62, 63].

### Multi-parameter FLIM

While lifetime and ratio indices are useful, multi-parameter combinations may yield even more useful data. Multispectral FLIM (ms-FLIM) applies to metabolism research based on NADH and FAD autofluorescence, which records FLIM images at multiple spectral bands. ms-FLIM is mostly applied in oral health research field, which was firstly adopted to distinguish the oral lesions from healthy tissue, as shown in Fig. 3a–iv [64]. With the development of ms-FLIM, it was applied to differentiate low-risk oral lesions, high-risk oral lesions, and oral carcinoma with high sensitivity and specificity in the hamster cheek pouch model [65]. The feasibility of ms-FLIM for early oral cancerous tissue detection was further testified by clinical trials [66, 67]. Besides, ms-FLIM dermoscopy system has been successfully constructed for discriminating basal cell carcinoma (BCC) from other dermatoses [68].

In addition to ms-FLIM, fluorescence lifetime analysis with other optic characteristics of substance to construct an improved classification model can also improve the accuracy of cancer diagnosis. For example, a three-dimensional classification model for differentiating glioblastoma (GBM) cells and metastasis cancer cells from control cells was constructed based on the redox ratio, second harmonic generation (SHG) intensity, and fluorescence lifetime, averaged for each tissue subgroup with a Gaussian ellipsoid fit [69]. Recently, another powerful method using a support vector machine (SVM) for classification based on the median, width, and entropy of lifetime τ_2_ was applied to breast cancer detection [70]. Similarly, as shown in Fig. 4c, by TCSPC-TP-FLIM scanning hematoxylin- and eosin-stained biopsy samples, the Gaussian distribution, Shannon entropy, and fluorescence lifetime were used to construct SVM classification systems for distinguishing BCC from actinic keratosis (AK) and Bowen's disease (BD) [71].

Table 2 presents a summary of this section.

**Table 2:** NAD(P)H & FLIM

Parameters	Comparative group			Result		Research goal	Refs.
FLT	Lung cancer tissue	Healthy tissue		<		Lung cancer tissue detection	[31]
	W31(cancer cells)	WFB		<		Cancer cell detection	[32]
	GSCs	NSCs		>		Metabolic profile change in GSCs	[36]
	GBM tissue	Normal mouse brain tissue		>		GBM tissue metabolic flux detection	[36]
	CSF-metastatic cell	Inflammatory cell		>		CSF-metastatic cell identification	[37]
	10%BSA on ice condition	Control		≈		Best preserved condition for cancer biopsy diagnosis	[44]
	Malignant thyroid lesions	Benign lesions	Healthy thyroid	>	≈	Malignant thyroid lesion detection	[33]
	BRCA2 mutation carrier	BRCA1 mutation carrier	Normal Lymphocyte cell	<	≈	High cancerous risk gene mutation cell line detection	[45]
NAD(P)H lifetime τ_2_	Glucose deprivation group	Control		>		Glucose deprivation in breast cancer cell detection	[50]
	LDH, PDK enzyme inhibition	Control		/		Key enzymatic steps inhibition monitoring	[51]
NADH/NAD +	Tumor cell	Monocyte/Macrophage cell		>		Tumor front delineation	[53]
FLIRR	Doxorubicin treatment	Pre-treatment		>		Doxorubicin treatment assessment	[62]
Free/bound NADH	HeLa	Fibroblast		↑	↓	Tumor-fibroblasts metabolism interaction	[49]
	Metastatic B16F10 melanoma	Pre-progression melanoma		>		Melanoma detection and staging	[54]
	Wnt/β signaling inhibition	Control		<		The relationship of Wnt/β signaling and tumor metabolic phenotype	[55]
	STIC	TMF		<		Classification of GBM subpopulation	[57]
	REV3L absence group	Control		<		A novel approach to minimize chemo-resistance of cisplatin treatment	[61]
	CIN1	CIN2	CIN3	≈	<	Cervical intraepithelial neoplasia grade	[56]
	ZsG	LN1	LN2	≈	>	Metastatic oral squamous carcinoma cell detection	[60]
	BCC	AK	BD	/		BCC, AK, and BD distinguishment	[59]
SVM	Adipose tissue	Fibrous tissue	Breast cancer tissue	/		Adipose, fibrous, and breast cancer tissue distinguishment	[70]
	BCC	AK	BD	/		BCC, AK, and BD distinction	[71]

"≈ <  > " represents absolute parameters value between adjacent two groups (from left to right); "↑ ↓" symbolizes parameter increase or decrease

*GSCs* Glioblastoma stem cells, *NSCs* Neural stem cells, *GBM* glioblastoma multiforme, *CSF* cerebrospinal fluid, *BSA* bovine serum albumin, *LDH* lactate dehydrogenase, *PDK* pyruvate dehydrogenase kinase, *STIC* stem-like tumor-initiating cells, *TMF* tumor mass-forming cells, *BCC* basal cell carcinoma, *AK* actinic keratosis, *BD* Bowen's disease

## FRET Combined with FLIM Applications

### Intracellular FRET-FLIM Applications

**Fig. 5:**
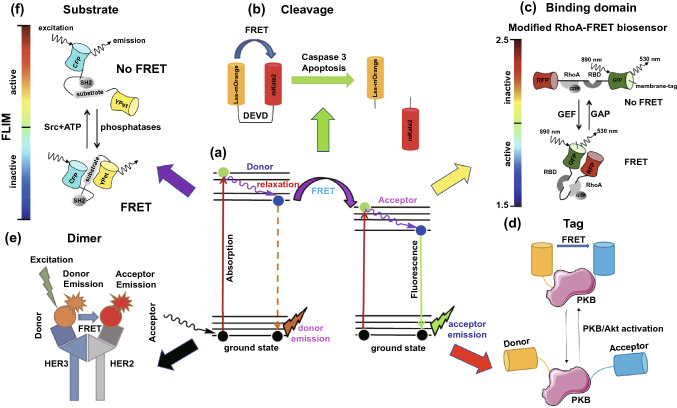
FLIM in combination with FRET for cancer diagnosis and treatment improvement. **a** Scheme of the FRET mechanism between an electron donor and receptor. Reproduced with permission from Ref. [75]. Copyright 2018, Elsevier; **b** Cleavage type: LSS-mOrange and mKate2 connected by an aspartate-glutamate-valine-aspartate (DEVD) linkage sequence for caspase-3 activity detection. Reproduced with permission from Ref. [77]. Copyright 2015, MDPI; **c** Binding domain type: Raichu-RhoA biosensor. Reproduced with permission from Ref. [80]. Copyright 2017, Elsevier; **d** Protein tag type: PKB tag biosensor for PKB activation state detection. **e** Dimer type: HER2-HER3 dimer for quantifying HER2-HER3 dimer. Reproduced with permission from Ref. [98]. Copyright 2016, Impact Journals; **f** Substrate type: FRET-based Src biosensor. The FLIM-bar indicates the Src activity from Src active (red to yellow) to inactive colors (green to blue). Reproduced with permission from Ref. [91]. Copyright 2014, Taylor & Francis. (Color figure online)

As an important energy transfer phenomenon, FRET has been applied in many research fields, especially in the protein interaction analysis [72, 73]. The basic principle of FRET is that when the distance between a donor and acceptor is less than 10 nm with an overlapped spectrum, favorable dipole–dipole interactions, and sufficient quantum yield, the excitation of the fluorescence donor molecule can cause the acceptor molecule to emit fluorescence, after which the fluorescence intensity of the donor decreases rapidly, as shown in Fig. 5a [74, 75]. Therefore, FRET efficiency increases with a decreasing donor fluorescence lifetime, which can be detected by a total internal reflection fluorescent microscope (TIRF) or FLIM.

In FRET-FLIM experiments, both fluorescent proteins (FPs) and organic fluorophores can be applied as fluorescent labels functioned as FRET pairs for protein–protein interaction analysis, where FPs were mostly reported as an element of genetically encoded biosensors [76]. Different types of protein activities require diverse types of FRET pairs for detection, which is exhibited in Fig. 5. The cleavage FRET pairs can be used to detect caspase-3 levels and treatment-induced apoptosis [77]. As shown in Fig. 5b, the active caspase-3 will cleave the DEVD sequence, resulting in LSS-mOrange and mKate2 separation, leading to a FRET efficiency decreases with the lifetime of the donor (LSS-mOrange) increases [78]. Additionally, a modified EGFP/mRFP Raichu-RhoA biosensor successfully observed the up-regulation of RhoA activity, which is related to numerous cellular activities and is known to drive cell motility in invasive cancers [79]. As shown in Fig. 5c, RhoA binds to GDP and exhibits non-FRET confirmation, whereas FRET confirmation appears when RhoA binds to GTP. Through FRET efficiency analysis, the activity of RhoA in tissue can be detected [80]. Various FRET biosensors for the detection of cancer-metabolism-related proteins, such as adenosine-monophosphate-activated protein kinase, have also been reported [81, 82]. Besides, cancers can also be induced by disturbed signal pathways. For example, in the FRET pairs in Fig. 5d, a high FRET efficiency between the labeling tags on protein kinase B (PKB) can be observed in the metastatic clear cell renal cell carcinoma sample, which is practical for predicting patient survival and optimizing personalized therapy. When the activation state of PKB/Akt increases, PKB will exhibit conformational change leading to FRET between the serine and threonine fluorescent labeling. The donor lifetime decreases, which can be recorded by FLIM [83]. In addition to the PKB/Akt signaling pathway, phospholipid signaling pathways are also involved in breast cancer. A novel phosphatidic acid (PA) biosensor called PASS has been successfully applied to the detection of the spatiotemporal production of PA, which is a type of phospholipid, based on decreased donor lifetimes [84]. Furthermore, posttranslational protein modification aberrations are also relevant to cancer occurrence and progression. Taking Poly(ADP-ribose)ylation (PARylation) as an example, PARylation can be monitored by using FRET-FLIM to assist in the PARylation dysregulation detection of cancer cells [85].

Regarding anti-cancer treatment, the capabilities of FRET-FLIM for drug validation have also been demonstrated [86]. For example, FRET-FLIM revealed the close distance between translation elongation factor eEF1A2 and an anti-tumor agent, plitidepsin, forming a drug-protein complexes in tumor cells [87]. Besides, for novel drug discovery and development, FRET-FLIM has been used to discover interaction domains between nuclear epigenetic integrators and their partners, as well as potential histone epigenetic therapeutic targets [88, 89]. Similarly, an unexpected sequence involved in the binding domain between anti-apoptosis and pro-apoptosis/BH3 proteins was found using FRET-FLIM, opening a new avenue for enhancing the combination of BH3 mimetic drugs and anti-apoptotic proteins for cancer treatment [90]. Additionally, for choosing superior treatment options, FRET-FLIM can provide important references by evaluating Src activity. As shown in Fig. 5f, once active Src phosphorylates the substrate, the biosensor will exhibit non-FRET confirmation. If dephosphorylation happens due to phosphatases, it will transform into the FRET confirmation [91]. In addition to these applications, a hot-FRET-based anti-cancer therapy called photodynamic therapy (PDT) also deserves attention. Based on material innovations, fluorescence lifetime imaging and PDT can be achieved in a single material, such as 3-aminophthalic acid [92]. Furthermore, this type of therapy can be improved through monitoring using sensitizer phosphorescence lifetime imaging and FLIM [93].

### FRET-FLIM for Cancer Cell Membrane Receptor Detection

In addition to protein interactions within cells, the receptors on membranes are also important biomarkers for cancer diagnosis. Traditionally, FLIM with a specific probe has been utilized to detect receptor expression levels, such as chemokine receptor 4 and human epidermal growth factor receptor 2 (HER2) [94, 95]. Recently, FLIM was combined with FRET pairs for membrane receptor detection. Among abundant receptors, the ErbB receptor family, including EGFR, HER2 and HER3, is considered to be one of the “superstar” families.

The ErbB receptor family participates in both signal conduction and cell proliferation and its abnormal content or activity is considered to be relevant to cancer initiation, progression, and treatment prognosis [96, 97]. By using FRET-FLIM, the HER2-HER3 dimer level has been detected by analyzing the fluorescence lifetime of HER3 fluorophore dye. As shown in Fig. 5e, FRET occurs between Alexa546 loaded onto HER3 (donor) and Cy5 loaded onto HER2 (acceptor), resulting in a decrease in the Alexa546 fluorescence lifetime [98]. Furthermore, the significant intra-tumor heterogeneity of epidermal growth factor receptor (EGFR) activity influences drug function. This has been monitored by novel FRET pairs of eGFP and mRFP1 based on the conformation change phenomenon because of Tyr phosphorylation using FLIM [99]. Similarly, by utilizing Alexa 546 and DyLight 649 labeled antibodies, FRET/FLIM has been applied to determine Gefitinib phosphorylation statuses for modulating the stability of EGFR dimers, providing a novel method for cancer-targeted therapy to deregulate EGFR signaling [100].

Previous studies on FRET-FLIM are summarized in Table 3.

**Table 3:** FRET & FLIM

Target molecule	FRET type	FRET pairs		FLIM	Potential application		Refs.
		Donor	Acceptor		Molecular	Clinical	
Trp	Dimer	Trp	NAD(P)H	TCSPC-FLIM	Bound NAD(P)H detection	Drug response evaluation	[63]
Caspase3	Cleavage	LSS-mOrange	mKate2	FD-FLIM	Apoptosis level detection	Drug evaluation	[77, 78]
RhoA	Binding domain	EGFP	mRFP-Raichu	TCSPC-FLIM	RhoA activity level detection	Invasive subtype identification	[80]
PKB	Tag	panAkt	pT308	FD-FLIM	PKB activation state detection	Personalized therapy	[83]
Phosphatidic Acid (PA)	Binding domain	GFP-tH/GFP-tK	RFP-PASS	FLIM	PA production and function detection	/	[84]
PARylation	Dimer	EGFP-ARTD1	TMR labeled NAD + analog	FLIM	PARylation level detection	Cancer diagnosis & Drug evaluation	[85]
eEF1A2	Dimer	DMAC	GFP	FD-FLIM	eEF1A2-plitidepsin interaction detection	/	[87]
UHRF1	Dimer	eGFP	mCherry	TCSPC-FLIM	/	MYST60 domain knockdown for cancer treatment	[88]
ER	Dimer	ALEXA488	ALEXA546	TCSPC-FLIM	Epigenetic therapeutic targets detection	Drug combination therapy	[89]
Bim	Dimer	mCer3	Venus	TCSPC-FLIM	/	BH3 mimetic drug improvement	[90]
Src	Substrate	ECFP	YPet	FLIM	Src activity detection	Drug combination strategy evaluation	[91]
HER2	Dimer	Alexa546	Cy5	TCSPC-FLIM	HER2-HER3 dimer level detection	Drug evaluation	[98]
EGFR	Substrate	eGFP	mRFP1	TD-FLIM	EGFR heterogeneity	EGFR targeted treatment improvement	[99]
EGFR	Tag	Alexa 546 F4	DyLight 649 F4	TCSPC-FLIM	Influence factor of EGFR dimer stability	Novel EGFR targeted anti-cancer therapy	[100]
EGFR	Dimer	Alexa 546 F4	Cy5 2F12	TD-FLIM	Exosomes' receptors heterogeneity detection	/	[121]

*FLIM* Fluorescence lifetime imaging microscopy, *TD-FLIM* time-domain FLIM, *FD-FLIM* frequency domain FLIM, *TCSPC-FLIM* time-correlated single photon counting FLIM

Footnotes: "FLIM" means the article did not mention specific time-domain or frequency-domain FLIM

## FLIM Combined with Fluorophore Dye Probes

### Intracellular Molecule Change Detection

Traditionally, the intensity method using fluorescent probes with FLIM has been very common. This method is based on over-expression receptors or molecules in cancer cells, such as Dmt-Tic-IR800 probes targeting overexpressed delta-opioid receptors in lung cancer cells and gold nanoclusters aimed at high glutathione levels in cancer cells [101, 102]. The fluorescence lifetime method is another promising technique for cancer diagnosis and treatment improvement.

**Fig. 6:**
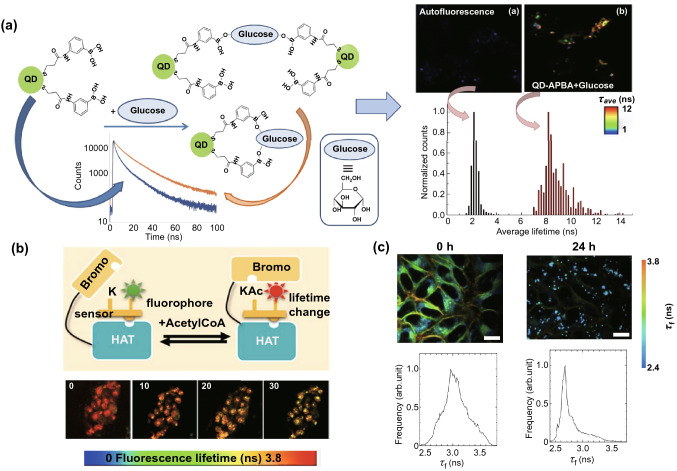
FLIM combined with a specialized probe for intracellular environment change detection. **a** Left panel: Schematic representation of the response when QD-APBA (quantum dot–aminophenyl boronic acid) conjugates encounter glucose. Right panel: FLIM imaging of MDA-MB-231 autofluorescence alone and under coexistence conditions, where MDA-MB-231 cells are incubated with 50 mM glucose and QD-APBA conjugates for 3 h. The corresponding photoluminescence lifetime histogram is presented below. Reproduced with permission from Ref. [108]. Copyright 2019, MDPI; **b** Upper panel: Sensor principle of the peptide biosensor. Lower panel: HATS acetylation FLIM image in a 3D culture over 25 min after treatment with 5 μM of anacardic acid (an inhibitor of PCAF acetylation activity), representing the capabilities of the peptide biosensor for acetylation level monitoring. Reproduced with permission from Ref. [106]. Copyright 2019, American Chemical Society; **c** FLIM images after HeLa cells are treated with oleic acid (400 μM) for 0 and 24 h. The cells were first co-cultured with PC6S (2 μM, 30 min, 37 °C). The corresponding fluorescence lifetime distribution histograms are presented (excitation wavelength: 488 nm, collected emission wavelength range: 510–560 nm. scale bar: 20 μm.). Reproduced with permission from Ref. [109]. Copyright 2020, American Chemical Society

In the nuclei of cells, the DNA binder, DAPI, has been combined with FLIM to detect two common aberrations in B-cell chronic lymphocytic leukemia (B-CLL) and acute lymphoblastic leukemia (B-ALL). Specifically, an extra 12^th^ chromosome and the deletion of p53 can be detected based on different lifetime changes [103]. Furthermore, a tripodal cationic fluorescent probe, called NBTE, has been combined with FLIM to detect G-Quadruplex. Based on the distinctive fluorescence lifetime response of G-Quadruplex, the content of DNA G-Quadruplex in lung, mammary and hepatic cancer cells is four times than that in normal cells, which can be exploited for cancer diagnosis [104]. Similarly, another G-Quadruplex fluorescent probe called o-BMVC foci, has also been used to identify G-Quadruplex motifs in combination with FLIM [105]. In addition to G-Quadruplex, the acetylation of histone lysine residues of acetyltransferase is another target for anti-cancer drugs that can be monitored by using a P300/CBP associated factor (PCAF) biosensor (HATS) in combination with FLIM (Fig. 6b). After the sensor binds to the histone acetyltransferase (HAT) target, upon acetylation, phosphor-lysine will be incorporated with the PCAF bromodomain, resulting in an increase of the fluorescence lifetime of the reporter [106].

Regarding research on the cytoplasm, the lifetime of the fluorescence dye ATTO425 on the aptamer AS1411 is shorter in cancer cells than that in normal cells [107]. Additionally, intracellular glucose levels can be detected based on the fluorescence lifetimes of aminophenyl boronic acid (APBA)-modified CdSe/ZnS quantum dots (QDs) (QD-APBA). As shown in Fig. 6a, when QD-APBA conjugates bind to glucose, there is an evident increase in lifetime compared to the control group, which can be detected by FLIM [108]. In addition to glucose consumption, Fig. 6c also shows that a π-extended fluorescent coumarin (PC6S) combined with FLIM can be successfully applied for lipid droplet imaging related to lipid metabolism [109]. As glucose and lipid metabolism are both relevant to cancer cells’ activities, these strategies are promising for cancer diagnosis and anti-cancer treatment efficiency elevation.

### FLIM-probe Applications to Cellular Membrane Structures

#### Membrane Potential

**Fig. 7:**
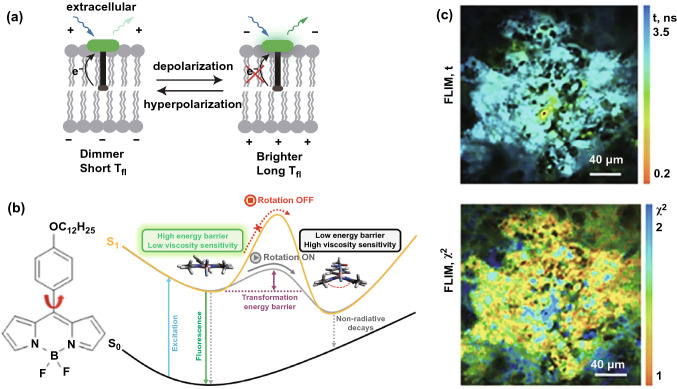
FLIM application to membranes for cancer diagnosis and treatment based on altered membrane potential and viscosity. **a** Mechanism of VoltageFluor dyes in which depolarization of the membrane potential attenuates the rate of photoinduced electron transfer. Reproduced with permission from Ref. [110]. Copyright 2019, elife Sciences Publications. **b** Left panel: Representative BODIPY molecular rotor. Right panel: Simplified mechanism of BODIPY rotors. Reproduced with permission from Ref. [114]. Copyright 2020, American Chemical Society. **c** Representative in vivo FLIM images of a CT26 tumor at 60 min after an intravenous BODIPY2 (3 mg kg^−1^) injection. The excitation wavelength is 800 nm and the detection wavelength is 409–680 nm. The upper FLIM image is used for lifetime/viscosity calibration and the lower image is a FLIM image of time-resolved fluorescence decay. Reproduced with permission from Ref. [117]. Copyright 2017, Springer Nature

In the field of studying membranes, a lack of non-invasive and sensitive tools for measuring absolute membrane voltage (*V*_mem_) restricts the understanding of the cellular activities behind cancer. Specific fluorescent dyed probes in combination with FLIM can be used to detect membrane potentials. For example, voltage Fluor dyes with FLIM have successfully detected membrane potentials based on longer lifetimes related to membrane potential depolarization, as shown in Fig. 7a, where one can see a positive linear relationship between lifetime and membrane potential [110]. Similarly, for the detection of mitochondrial membrane potential**,** green- and orange-emitting fluorescent dyes (SYTO) and tetramethylrhodamine methyl ester (TMRM) acting as FLIM probes have been applied to monitor mitochondrial membrane potential (MMP) changes, which was based on shorter lifetime related with enhanced mitochondrial polarization due to the quenching of fluorophores. The capability of these two fluorescent dyes for MMP detection was also testified in cancer cells and organoids [111]. Furthermore, the negative mitochondrial membrane potentials in cancer cells are important biomarkers that can be exploited to accumulate cytotoxic cationic molecules for anti-cancer treatment. The corresponding treatment outcomes can be analyzed using Phasor-FLIM [112, 113].

#### Membrane Viscosity

It is known that membrane viscosity affects membrane permeability, enzymatic activity, and other factors. Abnormalities are associated with malignant transformations, which can be detected by membrane viscosity probes mostly based on boron-dipyrromethene (BODIPY) structures, as shown in the left panel of Fig. 7b [114]. Based on the right panel of Fig. 7b, the fluorescence lifetimes of probes will increase under higher-viscosity conditions because the higher-viscosity leads to increasing transformation energy barrier between the planar conformation and the butterfly conformation of the BODIPY rotor, which impedes non-radiative decays.

In vitro, BODIPY in combination with FLIM has already achieved the quantitative measurement of viscosity in tumor cell spheroids and viscosity changes in cisplatin-treated tumor cells [115, 116]. To facilitate clinical applications, the improved fluorescent molecular rotors BODIPY1 and BODIPY2 have been combined with FLIM to accomplish in vivo viscosity monitoring. In an assay of viscosity imaging in CT26 subcutaneous tumors in vivo, Fig. 7c demonstrates that BODIPY2 rotors can be dislodged in a tumor, providing a sensitive and non-invasive method for detecting viscosity. For BODIPY time-resolved fluorescence decay, only mono-exponential decay is usable for lifetime/viscosity calibration, but biexponential results cannot be utilized because of the quenching of BODIPY aggregates. It is clear that mono-exponential decay is exhibited by the tumor cells and connective tissue, demonstrating that BODIPY2 is capable of in vivo viscosity imaging [117].

### FLIM Applications to the Extracellular Environments of Cancer Cells

**Fig. 8:**
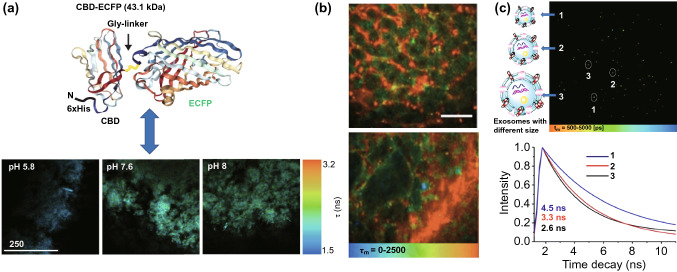
FLIM application to extracellular environments. **a** Schematic representation of a CBD-ECFP biosensor. The upper panel shows the CBD-ECFP structure, where N-terminal 6xHis sequences are shown in black with CBD and ECFP domains linked by Gly-rich linker regions. The lower panel shows fluorescence lifetime images of a CBD-ECFP GrowDex matrix at different pH levels (scale bar is 1 μm). Reproduced with permission from Ref. [118]. Copyright 2018, Elsevier; **b** Pseudo-colored fluorescence lifetime image produced by multiphoton fluorescence lifetime imaging microscopy. The image shows healthy liver tissue above and hepatocellular carcinoma below following QD injection (recorded at λExc/λEm: 900/515 to 620 nm; scale bar is 40 µm). Reproduced with permission from Ref. [119]. Copyright 2019, MDPI; **c** FLIM of exosomes of different sizes from tumor cells based on Mem-BDP probes. The upper and lower panels show FLIM images of exosomes of different sizes and their corresponding fluorescence decay curves, respectively. Reproduced with permission from Ref. [120]. Copyright 2019, American Chemical of Society

In combination with a well-designed fluorescent biosensor, FLIM can be utilized to monitor cancer milieu, such as the quantitative measurement of pH in engineered tissues based on the fluorescence lifetimes of novel biosensors, as shown in Fig. 8a, where a cellulose-binding domain (CBD) is fused with a pH-sensitive enhanced cyan fluorescent protein (ECFP) [118]. As cancer cells’ glycolysis metabolic trends leading to the reduced pH in cancer microenvironment, which reforms extracellular matrix for cancer metastasis, FLIM is promising for cancer progression and metastasis research. Additionally, the vascular morphology of tumor tissue is another important topic. An anionic ultra-small QD with an ultra-long fluorescence lifetime has successfully realized vascular imaging in normal and cancerous tissues in combination with FLIM. As shown in Fig. 8b, the neogenic vessels in tumor tissue are tortuous and disordered [119].

Outside of cells, exosomes, which exhibit number, size, and receptor heterogeneity, should not be neglected. Similar to cell membrane alteration detection, a rapid tumor diagnosis tool based on the combination of a membrane-targetable viscosity probe, called Mem-BDP, and FLIM, has been proposed to analyze membrane viscosity. Figure 8c demonstrates that larger exosomes originating from different cancer cells possessing lower viscosity result in a shorter fluorescence lifetime [120]. In addition to exosome size, the EGFR heterogeneity on oncosomes’ membrane can also be detected by FRET-FLIM based on the FRET occurrence between antibodies Cy52F12 on HER3 and Alexa 546 F4 on EGFR. Through this strategy, the oncogenic receptor signal rewiring was revealed which was helpful for optimizing anti-cancer treatments. [121] However, thus far, no methods based on the lifetime dimension have been reported for quantifying exosomes, meaning this area requires further investigation.

A summary of Sects. 5.1, 5.2, and 5.3 is presented in Table 4.

**Table 4:** Probe & FLIM

Aberration	Probe	Result	Potential application	Refs.
Extra chromosome 12	DNA binder: DAPI	FLT substantially increases	Diagnosis and classification for B-CLL and B-ALL	[103]
Deletion of p53		FLT mildly increase	Diagnosis and classification for B-CLL and B-ALL	[103]
G-quadruplex ↑	NBTE	G-quadruplex: cancer cells > normal cells	Differentiating tumor cells from healthy cells	[104]
Acetylation on histone lysine	HATS	FLT ↑	Detecting acetyltransferase-PCAF activity	[106]
Cytoplasm environment change	ATTO425 labeled on AS1411	FLT: tumor-5.8 ns control-4.3 ns	Differentiating tumor cells from healthy cells	[107]
Membrane potential change	VoltageFluor dyes	Membrane potential ↑ ~ FLT ↑	Detecting membrane potential change	[110]
	SYTO/TMRM	Mitochondria membrane potential↑ ~ FLT↓	Detecting mitochondria membrane potential change	[111]
Membrane viscosity change	BODIPY 2	Cisplatin treatment ~ viscosity↑ ~ FLT↑	Monitoring of the tumor response to anti-cancer therapy	[115]
	BODIPYC10/BODIPY + +	No viscosity difference in the center and periphery of spheroids	Non-invasive mapping of the viscosity of living tumor spheroids	[116]
	BODIPY 1/BODIPY 2	Viscosity imaging in CT26 subcutaneous tumors in vivo **√**	In vivo monitoring of viscosity of tumor	[117]
pH change	CBD-ECFP	pH↑ ~ FLT↑	Detecting pH in the tumor microenvironment	[118]
Vascular disorder	QDs	Tortuous vascular in tumor tissue	Imaging of vascular based on QDs	[119]

### Fluorescence Anti-cancer Drugs with FLIM for Drug Delivery Research

**Fig. 9:**
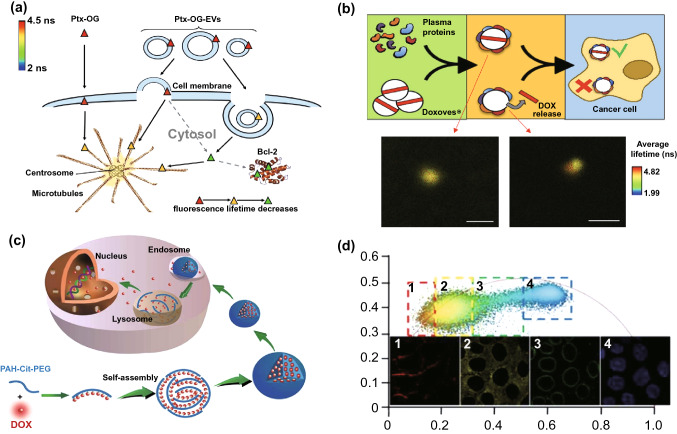
Fluorescent anti-cancer drug in combination with FLIM for drug delivery and treatment improvement. **a** Drug entry pathway: Scheme of OG fluorophore-dyed Ptx and EV-mediated Ptx-OG cell entry pathways. Different sizes of vesicles represent the EV size heterogeneity considered in this study. The colors of Ptx-OG in the figure (red, yellow, green) are consistent with the FLIM image color scale. Solid arrows represent guaranteed pathways of Ptx-OG and Ptx-OG-Evs into the cell, while dashed arrows represent presumed pathways required for proving effectiveness. Reproduced with permission from Ref. [123]. Copyright 2018, Elsevier. **b** Drug delivery outside a cell: scheme of the dynamic BC formation of liposome DOX, determining the success or failure of liposomal drugs. Reproduced with permission from Ref. [124]. Copyright 2018, American Chemical of Society. **c** Scheme of a DOX-loaded nanostructure and drug release in a cell triggered by a lower pH. Dox was covalently loaded onto a pH-sensitive polymer (poly(allylamine)citraconic anhydride, PAH-Cit) for the pH-sensitive release of Dox. The entire process was monitored by Phasor-FLIM. Reproduced with permission from Ref. [129]. Copyright 2018, American Chemical Society. **d** Intracellular drug distribution: Phasor-FLIM of MCF-7 cells incubated with PAH-Cit/DOX after 6 h. Phasor plot of FLIM images shown above. Reproduced with permission from Ref. [129]. Copyright 2018, American Chemical Society. (Color figure online)

Fluorescence drugs or fluorescent-dyed drug carriers combined with FLIM are useful tools for drug delivery. In a previous study, emission wave changes in drug fluorescence caused by conformational alteration were applied to analyze delivery processes [122]. However, the fluorescence wave changes indicated by light color shifts were not as sensitive as desired, but fluorescence lifetime may be a promising solution for sensitivity improvement. For example, different entry pathways for exosomes (EXOs) and microvesicles (MVs) can be investigated using the method shown in Fig. 9a. By using Oregon green (OG)-labeled paclitaxel (Ptx) loaded onto EXOs and MVs, and based on fluorescence lifetime differences, FLIM can determine that exosomes deliver drugs via endocytosis, whereas most microvesicles enter cells via both endocytosis and fusion with cell membranes [123].

Liposomal-based anti-cancer drug delivery systems are capable of delivering fluorescent anti-cancer drugs such as doxorubicin and curcumin using FLIM for process monitoring. As shown in Fig. 9b, the first obstacle to liposome drug delivery is dynamic biomolecular corona (BC) formation in vivo, which changes the original liposome synthetic identities, leading to drug leakage. While Doxoves® was selected as a control group following exposure to human plasma (HP), Doxoves® was surrounded by complex BC that changed the liposome's synthetic identity. The corresponding FLIM images of Doxoves® (left panel) and Doxoves®-HP complexes (HP = 50%, right panel) in a 1.7% agarose gel (bottom two images) demonstrated that the failure of DOX delivery was relevant to the rupture of the membrane and drug release in a physiological environment [124]. Furthermore, FLIM has also been employed to investigate the mechanisms behind the low nuclear internalization efficiencies of drug delivery patterns such as liposomal doxorubicin (L-Dox), thereby increasing their clinical utility [125].

In addition to liposomal-based methods, numerous novel nanomedicine delivery systems have also been constructed, where FLIM mainly functions as an auxiliary monitoring apparatus [126]. An experiment using free Dox and Au-Dox was performed on melanoma B16 cells and cardiomyocytes. FLIM revealed that while Au-Dox entered the nuclei of the B16 cells, only free Dox could enter the nuclei of the cardiomyocytes, indicating that Au-Dox has reduced toxicity to normal cells [127]. Another promising DOX delivery system, called DOX-loaded graphene oxide (GO) or GO-DOX, has exhibited much higher anti-cancer efficiency than L-DOX. FLIM determined that the mechanism behind this improvement was that GO-DOX bound to cell plasma membranes to induce massive intracellular DOX release [128]. Additionally, a novel DOX delivery system, called PAH-Cit-Dox, has also been constructed, as shown in Fig. 9c. The DOX release and intracellular distribution were analyzed by Phasor-FLIM and phasor-differentiated lifetime pixel intensity in Fig. 9d. Based on the diverse fluorescence lifetimes of DOX, MCF-7 cells were separated into four sections of cell membranes, cytoplasm, nucleus membranes, and nucleus in the phasor-differentiated lifetime pixel intensity image [129]. Similarly, a novel pH-sensitive nano-chemotherapeutic system, called AuNPs@gelatin-hyd-Dox, was synthesized for tracking the release and intracellular kinetics of Dox and Au-NPs by detecting fluorescence signals and photoluminescence using TP-FLIM [130]. Furthermore, FLIM has successfully detected a novel hybrid probe internalization pathway used for drug delivery (consisting of β-D-glucan and the boronic acid derivative coumarin), as well as drug distributions inside cells [131]. Generally, fluorescent drugs combined with FLIM are promising for drug delivery optimization, which can lead to better anti-cancer treatment outcomes.

## Conclusion and Outlook

In general, according to its development history from autofluorescence molecules to fluorescent dyed probes and from intracellular substances to extracellular environments, FLIM has been widely applied to cancer diagnosis and treatment monitoring for further improvement. The applications of NAD(P)H-FLIM for metabolism, FRET-FLIM for molecular interaction, and fluorescent dyed probes aimed at specific aberrations all demonstrated FLIM's great potential for cancer research. With the development of new optical instruments and analysis methods, as well as cancer biomarker discoveries, the application of FLIM has broadened, and a rapid increase in FLIM-assisted clinical cancer diagnosis and tumor boundary determination cases have been reported. Additionally, as novel abnormities in cancer cells are uncovered, these discoveries can lead to anti-cancer drug innovations. Despite these inspiring achievements so far, FLIM still has some shortcomings that must be addressed.

For realizing FLIM clinical application, there are numerous techniques for increasing imaging speed and resolution, which were discussed in detail in the introduction section. In particular, for faster FLIM, several commercial solutions are available for realizing imaging at kilohertz rates. For example, an apparatus from Becker & Hickel can speed up imaging using an improved femtosecond laser and laser scanning vibration mirror, whereas the PicoQUant company has combined time-tagged and time-resolved data handling with a picosecond or femtosecond pulsed laser and single-photon detector for faster imaging. In addition to improvements of FLIM itself, FLIM has also been increasingly integrated with a variety of optical techniques to gain a more comprehensive understanding of cancer cells based on novel nanospheres and optical systems [132, 133]. For example, for metastatic pancreatic cancer detection, a FLIM system containing a super-wide-tuning integrated multimodal platform for label-free evaluation has been constructed to collect detailed cell and milieu information [134].

Besides, as most bio-life researches are conducted in cell experiments or animal models, several issues are needed to be addressed from bench to bedside. Considering the potential toxicity of organic fluorophores or FPs, the biocompatibility and biodegradability of FRET pairs or fluorescent biosensors are primary problems that should be solved. Moreover, for in-vivo applications, the targeting ability to cancerous regions of biosensors for FLIM detection is vital for efficiency. Furthermore, most clinical in-vivo applications are restricted in skin, oral health, or tract surface because of the imaging depth limitation of traditional fluorophores. To solve this problem, near infrared (NIR) fluorophores, whose excitation and emission wavelengths are within 650–900 nm, overcome the absorbance disadvantages, greatly elevating the penetration depth to centimeters [135]. These NIR fluorophores greatly broaden the application fields, which especially deserve medical investigator’s attention.

**Fig. 10:**
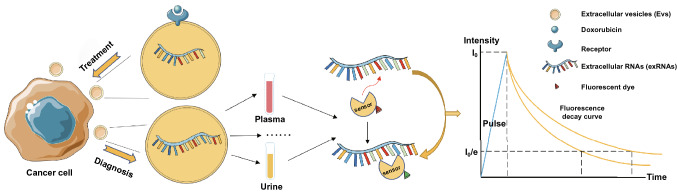
Scheme of exosome based FLIM potential application for cancer diagnosis and treatment monitoring. This novel treatment strategy is based on the co-delivery of fluorescent anti-cancer drugs such as doxorubicin with exRNAs. The cancer diagnosis strategy is based on the targeted exRNA detection of oncosomes. In this diagnosis method, fluorescent-dyed biosensors identify the specific base sequences of exRNAs in body fluids, which will exhibit fluorescence lifetime changes that can be detected by FLIM

To further broaden the application region of FLIM, FLIM in combination with NAD(P)H for metabolism change detection can be adopted not only in cancer cells, but also in adjacent cells, such as immune cells for improving immunotherapy and neuron cells for deepening our understanding of cancer neuroscience studies [136, 137]. Additionally, extracellular vesicles (EVs) based method can combine with FLIM to play a significant role in both diagnosis and treatment monitoring. FLIM is hopeful for specific exRNA detection based on novel RNA probes targeting specific RNA sequences. As shown in Fig. 10, if a probe binds to a particular RNA sequence, the corresponding fluorescence lifetime can be analyzed by a computer. As high-throughput sequencing continues to develop, optimized FLIM probe arrays should enable the rapid and sensitive exRNA detection of oncosomes, which could surpass traditional sequencing techniques. Regarding anti-cancer treatment, by using EVs to deliver RNAs, such as long noncoding RNAs and circular RNAs, to proximal and distant recipient cells to avoid the RNAase effect, exRNA-loaded EVs can achieve better treatment outcomes [138, 139]. Considering the stable presence of extracellular RNAs (exRNAs) in EVs, EVs can load transfected specific RNA such as siRNA, miRNA, and fluorescence anti-cancer drugs based on FLIM monitoring. Such EVs are promising for improved anti-cancer targeted therapy and the entire delivery process can be precisely tracked by FLIM. Although extracellular vesicle-mediated RNA delivery has been demonstrated previously, combining fluorescent drugs and exRNA with FLIM for cancer therapeutic effect improvement has recently been proposed for the first time [140]. Collectively, the researches up to now have demonstrated the potential of FLIM as a promising tool for cancer diagnosis and treatment monitoring, and future studies, including FLIM optimization and fluorophore improvement, are warranted for its efficacy improvement on clinical applications.
